# Efficacy of aerobic exercise for HIV-associated neurocognitive disorders receiving ART: An RCT

**DOI:** 10.4102/sajp.v80i1.2104

**Published:** 2024-12-11

**Authors:** Martins Nweke, Nombeko Mshunqane

**Affiliations:** 1 Department of Physiotherapy, Faculty of Health Sciences, University of Pretoria, Pretoria North, South Africa; 2 Department of Physiotherapy, Faculty of Health Sciences, David Umahi Federal University of Health Sciences, Uburu, Nigeria

**Keywords:** HIV infection, HIV-associated neurocognitive disorder, aerobic exercise, randomised controlled trial, activity and participation limitation

## Abstract

**Background:**

HIV-associated neurocognitive disorder (HAND) affects an individual’s capacity for independence and engagement in everyday activities, posing challenges in environments with limited resources and low social support.

**Objectives:**

To ascertain the efficacy of exercise (AE) for activity and participation (AP) level in people with HAND.

**Method:**

This is a randomised controlled trial that is parallel-group in nature, with intention-to-treat analysis and disguised allocation. Seventy-three people with HAND in total were randomised at random to the AE and control groups. Three 20–60-min sessions of moderate-intensity AE training on a cycle ergometer were included of the 12-week intervention. Individual tolerability served as the basis for progression. Three months after the intervention, at the conclusion of the intervention, and at the baseline, outcomes were measured. The AP constitutes the main outcome variable. To investigate the impact of AE on AP level, rank analysis of covariance was performed after log-transformation.

**Results:**

When comparing the AE to the control group, there were significant increases in social wellbeing AP (Cohen *d* = 0.550; *p* = 0.021), emotion AP (Cohen *d* = 0.641; *p* = 0.007) and overall AP level (Cohen *d* = 0.896; *p* < 0.001). There was no discernible variation in AP across the groups three months following AE (Cohen *d* = 0.437; *p* = 0.067).

**Conclusion:**

AE induces a small increase in AP among individuals with HAND.

**Clinical Implications:**

For people with HAND, regular AE is a good way to manage AP restriction. Increasing AE participation may improve AP restriction.

## Introduction

Human immunodeficiency virus (HIV) survivors live longer when receiving highly active antiretroviral therapy (HAART) (Marcus et al. 2016; May et al. 2014). But HAART comes with its own set of drawbacks, such as the ongoing cost of antiretroviral therapy (ART) and the various health issues related to HIV (Olivier, Cacabelos & Naidoo 2018; Thakur et al. 2019). Perhaps the most well-known neurologic effect of HIV infection is the HIV-associated neurocognitive disorder (HAND) (Dubé et al. 2005). Patients often experienced severe cognitive impairment prior to the widespread use of HAART, which was linked to frailty, falls and sarcopenia (Ances & Ellis 2007). However, in the present period, the prevalence of HIV-associated dementia has declined dramatically, with very few cases documented among individuals living with HIV (PLWH) (Anthony & Bell 2008). In spite of this, low- and middle-income countries (LMIC) have shown an increase in the frequency of HAND (Wang et al. 2020). Two important aspects of the pathophysiology of HAND include early brain HIV infection and HAART’s failure to effectively cross the blood-brain barrier. The brain continues to serve as a breeding ground for HIV replication even when systemic viral suppression is achieved (Saylor et al. 2016). It’s important to note that HAND still exists in both wealthy and developing nations, with Sub-Saharan Africa and other LMICs seeing the greatest effects (Nweke et al. 2021a; Wang et al. 2020).

Although the resulting neurocognitive deficits differ from person to person, memory loss, impulsivity, impatience, visuospatial issues, acalculia and difficulties focusing and paying attention are among the more typical signs (Clifford & Ances 2013; Modi, Mochan & Modi 2018). Studies on HIV-positive persons have shown a correlation between cognitive impairment and functional constraints as well as participation limitations (Chernoff et al. 2010; Gouse et al. 2020; Marquine et al. 2018; Schifitto et al. 2001; Thames et al. 2011). In everyday life, functional independence is essential for survival, particularly in environments with few resources and low social support. Moreover, HAND has a detrimental influence on mental health, hastening ageing and raising risk-taking behaviours related to health, which hinders effective engagement (Anand et al. 2010). This puts at risk attempts to stop the spread of HIV and remove it from the list of illnesses associated with poverty (Anand et al. 2010). Therefore, HAND needs to be considered while attempting to reduce the socioeconomic burden of HIV infection among those living with HIV.

One effective method for reducing HAND is the widespread prescription of antiretroviral medication (ART) (Force et al. 2021). Many people have chosen to supplement ART with different pharmaceutical therapy, such as intranasal insulin, cannabinol and psychostimulants, despite the fact that ART alone is insufficient to eradicate HAND (Singer & Thames 2016). For moderate types of HAND that self-limit, there is currently no effective treatment.

It has been found that incorporating physical exercise interventions into HIV/AIDS management programmes improves a number of health outcomes (Aucamp 2008; Furler et al. 2003). Maintaining one’s functional status through regular physical activity includes the ability to do activities of daily living (ADL) and participate in social activities (Paterson & Warburton 2010). In addition, consistent exercise enhances functional and physiological characteristics and lowers the likelihood of mobility impairments over time (Paterson & Warburton 2010). More precisely, according to Rosenoff et al. (1999; Dudgeon et al. 2004), aerobic exercise training may enhance physical functioning in PLWH (Dudgeon et al. 2004).

According to Jaggers et al. (2015) and Clayson et al. (2006), aerobic exercise training is generally safe and enhances lean muscle mass, muscular strength, cardiorespiratory fitness, metabolic profile and overall quality of life in people with Parkinson’s disease. Given the complexity and devastating nature of HAND, it is unclear whether or how regular physical activity encourages participation in everyday activities and activity in individuals with HAND diagnoses. Thus, the purpose of this study was to ascertain how aerobic exercise affected the engagement and activity levels of people with HAND. This paper is a component of a randomised controlled study investigating how aerobic exercise affects the prevalence of HAND.

## Research methods and design

### Trial design and registration

This was a parallel randomised control trial (RCT) with 1:1 allocation made possible by a restricted assignment scheme. Aerobic exercise was the intervention, and a control group without access to aerobic exercise served as the comparator. The trial was registered with the PAN African Trial Registry (PACTR) (ID: PACTR202009483415745).

### Participants

People with HAND diagnoses were included in the study population. The following were employed as inclusion criteria: formal education (at least primary 6), HAND diagnosis, physical inactivity (sedentary or 2 h per week) and readiness to exercise upon evaluation (had not engaged in regular exercise for around 3 months prior to the study). Participants who were 65 years of age or older had uncontrolled hypertension (BP 140/90 mmHg), a history of focal neurological deficit, psychiatric illness or traumatic brain injury, substance abuse, alcoholism, depression, recent musculoskeletal injuries, significant deafness, eye impairment, pregnancy, angina pectoris, shortness of breath, active use of nootropic supplements, cognitive enhancer or medication for attention-deficit/hyperactivity disorder were excluded from the study.

The University of Nigeria Teaching Hospital (UNTH) served as the study’s location. Based on preliminary data, the UNTH ART clinic’s potential participants were primarily from Enugu Metropolis, making up almost half of the total. This idea led to the selection of the second venue, the Notch Physiotherapy Clinic, as a more convenient location for participants travelling from Enugu Metropolis and the surrounding areas. The trial locations were comparable in terms of convenience. The intervention team (two qualified physiotherapists and two trained research assistants) was trained by the principal investigator to ensure uniformity.

### Intervention

The aerobic exercise group cycled on ergometers at 60% – 80% of their maximum heart rate, following ACSM guidelines (Garber et al. 2011). This 12-week program involved three weekly sessions. Because of the chronic nature of HIV, sessions in the first month lasted 20–30 min based on individual fitness levels, then increased to 30–45 minutes and reached 60 min by the eighth week. Participants aimed for moderate intensity, with certified physiotherapists overseeing preparation and evaluations as per ACSM standards (Garber et al. 2011).

The control group received educational information at the start and midpoint of the study, including messages about the benefits of exercise for people living with HIV. They were instructed to avoid exercise until further notice. ART adherence was measured by comparing taken to prescribed medications, while exercise adherence was the ratio of attended sessions to the 36 expected sessions.

After 4 weeks, training sessions lasted between 30 min and 45 min, and after 8 weeks, they lasted 60 min each. It was recommended that participants give the moderate-intensity workout their all. Before beginning, every participant complied with the ACSM guidelines (Walker et al. 1992). Qualified physiotherapists performed all fitness evaluations. At the beginning of the trial and at the conclusion of the sixth week, the participants in the control group consented to receive text messages with information about the health advantages of physical activity. Furthermore, this group was asked to refrain from exercising in any way until instructed to do so by the research team.

### Exercise testing

Exercise testing was conducted following frequency, intensity, type and time (FITT) principle. The frequency was thrice per week; the intensity was moderate (60% – 80% of HRmax). The type was bicycle ergometer the Life-Fitness Cycle Ergometer (95Ci, US), and the time was 20–60 min per session. The life-Fitness Cycle Ergometer (95Ci, US) is a high-quality fitness machine that providing an efficient and comfortable cardiovascular exercise. Exercise testing was conducted at both baseline and after the twelfth week using the Young Men Christian Association (YMCA) bicycle ergometer protocol (Ezema et al. 2020; Walker et al. 1992). The YMCA protocol is a submaximal bicycle ergometer test that uses heart rate to estimate oxygen consumption (VO2). Since its inception, it has become a widely used indirect method for predicting VO2max. Participants pedal at a steady pace through stages with increasing workloads. The protocol effectively predicts VO2max in the general population (Beekley et al. 2004; George et al. 2000) and among physically active individuals (Kovaleski et al. 2005). The YMCA protocol called for two to four 3-min continuous activity bouts and two HR-power output data points (steady-state heart rate: HR) in a range of 110–150 bpm. The test’s objective is to increase the participant’s heart rate steadily to 110–150 beats per minute, or 80% of their age-predicted maximum heart rate (HRmax), for a minimum of two phases in a row. The first three minutes of exercise on the Life-Fitness Cycle Ergometer (95Ci, US) were set between 25 and 50 watts at a speed of 50 rpm.

Once an HR of greater than 110 bpm was reached in the first three minutes at 75 watts, only one more three-minute stage at 450 kg.m.min-1 was needed. However, to produce two HR varying between 110 and 150 beats/min, two further stages were needed at workloads ranging from 75 to 125 watts, with the second stage HR being 110 beats/min. On the YMCA graph sheet, the two steady-state HRs were plotted against their corresponding effort. A perpendicular line to the x-axis was drawn to estimate the work rate (maximal oxygen consumption or VO2max), which would have been reached if the person had worked to their full capacity. The line produced from the plotted points was then projected to the age-predicted HRmax (Ezema et al. 2020; Lamina & Okoye 2011; Walker et al. 1992).

### Outcomes

The primary outcome – activity and participation restrictions – was assessed using the Oxford Activity and Participation Questionnaire (OX-PAQ). Secondary outcomes were ART adherence, exercise adherence (number of sessions attended compared to the 36 total planned sessions), pulse rate and systolic and diastolic blood pressure. At the beginning of the exercise programme, at its conclusion and 3 months after the intervention, all variables were evaluated. Depending on the distribution of the data, an aggregate parameter such as a proportion, mean or median was employed. Under the direction of a neurologist, results were evaluated by a clinical psychologist and physiotherapist.

#### Activity and participation

Activity and participation restrictions were evaluated using the OX-PAQ (Morley et al. 2016). The Ox-PAQ is a 23-item patient-reported outcome questionnaire that measures physical activity and participation. It is used in clinical trials to assess the efficacy of therapies meant to increase involvement and activity. It is theoretically based on the World Health Organization (WHO) International Classification of Functioning, Disability, and Health (WHO 2001). According to Kelly et al. (2016), the Ox-PAQ has a low rate of missing data and strong reliability (Cronbach’s alpha: 0.81–0.96) and validity across all three domains. Additionally, it demonstrates strong convergent validity when compared to the 5-dimensional EuroQol questionnaire (Herdman et al. 2011). In accordance with Jenkinson et al. (2019), the Ox-PAQ was used to generate a single score by summing the three domains and expressing the outcomes on a scale of 0 to 100. Higher scores indicate more challenges with involvement and activities.

#### Physical activity readiness

Physical activity readiness was assessed using the Physical Activity Readiness Questionnaire (PAR-Q). The PAR-Q was developed by the British Columbia Ministry of Health in partnership with the Multidisciplinary Board on Exercise (Warburton et al. 2011). It is used to develop an exercise programme and help assess a person’s fitness level by evaluating the safety or possible risk of exercise in relation to their medical history, present symptoms and risk factors. Before enrolling patients in the clinical trial, the measure was used to assess the patient’s physical activity readiness (Whitfield et al. 2014).

#### Cardiovascular parameters

The individuals’ resting heart rate (HR), systolic blood pressure (BP) and diastolic blood pressure (BP) were measured using the Accoson Sphynomanometer (A.C. Cossor & Son (SURGICAL) LTD., London, England) and Littman Stethoscope (3M Health Care, Eden Prairie, US). Every day from 7:00 a.m. until 2:00 p.m., all cardiovascular assessments were completed.

### Sample size

We utilised power analysis to obtain the required sample size. Using a Cohen medium effect size of 0.7, a power of 0.8, and a degree of freedom of 1, we obtained a sample size of 68 (34 in each group). The G-power version 3.1.9.7 (Faul et al. 2009) was used to aid the power analysis. The level of significance was set at α = 5%. To select the study participants, a random sampling was utilised as described below.

### Randomisation

Using Random Restricted Software 2.0, a series of random numbers was generated (Saghaei 2004). Restrictive randomisation with a block size of four was applied. The letter-size opaque brown sealed envelopes with the letters ‘C’ or ‘E’ written on the edge were used to seal the random generated numbers. The letters ‘C’ and ‘E’ represented ‘control’ and ‘exercise’, respectively. Participants were asked to pick one envelope from the pool and handover to a research assistant who opened the envelope and registered each subject in the selected group. Allocation of participants into control and treatment groups was carried out by a trained research assistant. Also, the random sequences were generated by a trained research assistant.

### Statistical analysis

We replaced missing data with multiple imputations and performed an intention-to-treat analysis. The multiple imputation was necessary to aid the intention-to-treat approach, which is recommended to preserve the benefits of randomisation. The distribution of continuous data was ascertained using the Shapiro-Wilk test, and the data were improved by log-transformation. A chi-square test was performed to compare baseline category outcomes between groups. We utilised Quade’s ANCOVA test and rank analysis of covariance (ANCOVA) to assess post-treatment outcomes between groups. The statistics used in our analysis were mostly non-parametric but suitable for our data distribution. Hence, the inference is void bias resulting from inappropriate statistical computations. Within-group comparisons were determined using the Friedman test. Version 22 of the Statistical Package for Social Sciences (SPSS) was used to analyse all of the data. Lastly, using the F-value that was found in the statistics after Lenhard and Lenhard (2015), we calculated Cohen *d*, which measures the size of the effect of the intervention on the outcomes. This was necessary to aid intuitive interpretation of the comparison of the outcomes between the control and exercise groups.

### Ethical considerations

Ethical clearance to conduct this study was obtained from the University of Pretoria’s Faculty of Health Sciences’ Research Ethics Commitment granted ethical approval (reference no. 152/2020).

## Results

**FIGURE 1:**
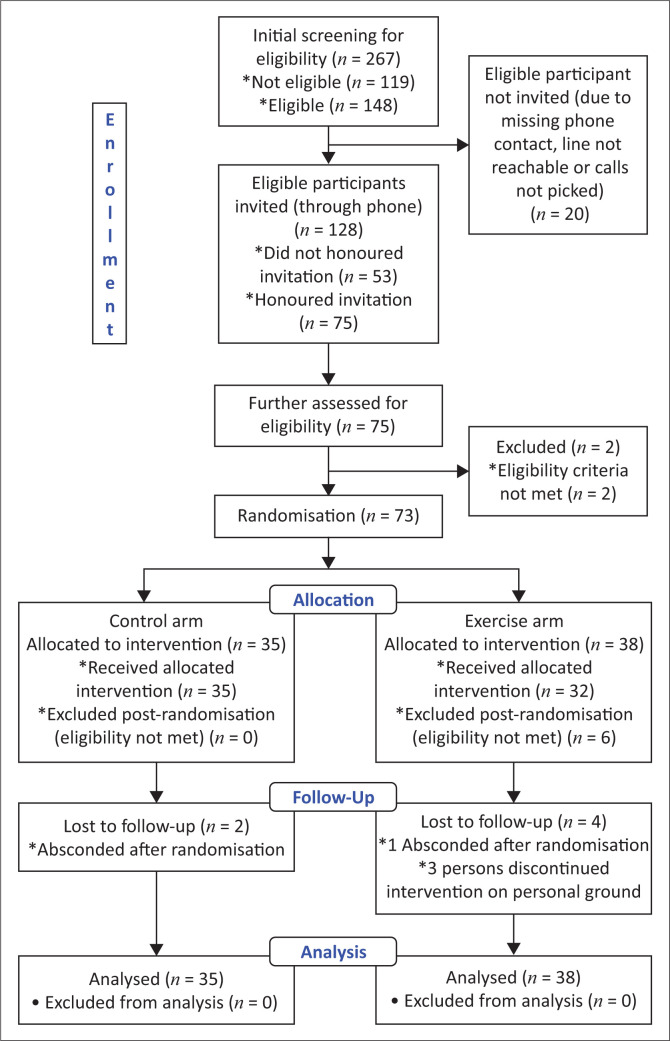
CONSORT Flow Diagram: The consort flow diagram describing the participants’ selection process.

Only 75 of the 128 individuals deemed eligible and invited accepted the invitation. Only 73 of the 75 individuals satisfied the post-screening criteria for eligibility for pre-exercise. After that, eligible individuals were randomised at random to either the exercise (38) or control (35) arms. Six participants were eliminated once more after a post-randomisation review. Four individuals in the exercise arm and two in the control arm were lost to follow-up. All randomly assigned individuals were included in the final analysis through the use of the intention-to-treat analysis (Figure 1).

The subjects’ cardiovascular and sociodemographic details have already been published (Nweke et al. 2021b). In this work, we restate these findings because they guide our statistical analysis and discussion. Age (*p* = 0.887), gender (*p* = 0.704) and educational attainment (*p* = 0.744) did not significantly differ between the exercise and control groups at baseline. At baseline, there were significant differences in the diastolic blood pressure (*p* < 0.001), pulse rate (*p* = 0.001) and ART adherence rate (*p* = 0.001) (Nweke et al. 2021b). In comparison to the control group, the exercise group scored considerably higher in the social connection QoL (*p* = 0.009) and overall QoL (*p* = 0.0028) (Table 1).

**TABLE 1:** Comparison of baseline clinical characteristics and levels of activity and participation between experimental and control groups.

Approach	Intention-to-treat				Per protocol			
	Exercise		Control		Exercise		Control	
	MR	MR	MWU	*p*	$\bar{\text{x}}$(s.d.)/MR	$\bar{\text{x}}$(s.d.)/MR	*t*/MWU	*p*
Routine activity	180.86	180.11	620.00	0.945	44.6 (10.29)	46.46 (6.88)	−0.85	0.400
Social wellbeing	166.14	190.20	523.65	0.009*	33.14	38.94	527.00	0.160
Emotion	189.27	171.23	560.19	0.098	13.47 (4.16)	13.17 (3.44)	0.33	0.741
Overall AP	181.53	179.41	615.29	0.028*	71.50(16.72)	74.11 (10.32)	−0.79	0.432

MR, mean rank; MWU, Mann-Whitney U test; $\bar{\text{x}}$ (s.d.), Mean (standard deviation); *t, t*-statistic; AP, activity and participation.

* , StatistIcally significant at alpha = 0.05.

After completing a 12-week aerobic exercise programme, the exercise group’s scores on the emotional wellbeing (Cohen *d* = 0.641; *p* = 0.007), overall AP level (Cohen *d* = 0.896; *p* < 0.001) and social wellbeing (AP) were all significantly higher than those of the control group. Nevertheless, there was no discernible change in activity and participation between the exercise and control groups at the conclusion of the 3-month follow-up (Cohen *d* = 0.437; *p* = 0.067) (Table 2). At the conclusion of the follow-up, we saw the highest activity and engagement levels within each exercise (*p* < 0.05) and control group (*p* < 0.05) (Table 3).

**TABLE 2:** Comparison of post-treatment clinical characteristics and levels of activity and participation between exercise and control groups.

Approach	Intention-to-treat				Cohen *d*	Per protocol			
Variables	Exercise MR(s.e.)	Control MR(s.e.)	*F*	*p*		Exp $\bar{\text{x}}$(s.e.)/MR	Control $\bar{\text{x}}$(s.e.)/MR	*F*	*p*
**Immediate post-treatment**									
Routine Activity	134.94† (7.08)	122.51† (7.36)	1.479	0.225	0.289	27.85	26.12†	328.00	0.681
Social wellbeing	64.17† (0.30)	16.62† (0.31)	5.365	0.021*	0.550	31.37	22.46	233.00	0.035*
Emotion AP	18.65† (0.42)	16.69† (0.44)	7.272	0.007*	0.641	26.91	27.10	348.50	0.962
Overall AP	83.45† (1.05)	76.53† (1.11)	14.212	< 0.001*	0.896	29.24	24.67	290.50	0.281
**Follow-up**									
Routine Activity	62.24† (0.56)	60.33† (0.60)	3.740	0.054	0.459	33.31	27.50†	362.50	0.187
Social wellbeing	19.24† (0.18)	18.73† (0.19)	2.671	0.103	0.388	20.89† (3.33)	19.76† (3.19)	1.318	0.193
Emotion AP	20.35† (0.28)	19.99† (0.30)	0.508	0.476	0.169	32.94	27.90	374.00	0.131
Overall AP	88.49† (0.71)	86.20† (0.76)	3.387	0.067	0.437	34.24	26.50	333.50	0.085

MR, mean rank; MR (s.e.), mean rank (standard error); MWU, Mann-Whitney U test; $\bar{\text{x}}$(s.e.), mean (standard error); AP, activity and participation.

* , Statistically significant at alpha = 0.05.

† , Adjusted based on baseline outcome.

**TABLE 3:** Within-group comparison of clinical characteristics and levels of activity and participation level.

Approach	Intention-to-treat					Per protocol				
Variables	Mean rank			χ^2^	*p*	$\bar{\text{x}}$(s.d.)			*F*	*p*
	Base	Post-Rx	Follow-up			Base	Post-Rx	Follow-up		
**Within exercise group**										
Routine Activity	1.17†	2.17†	2.66†	226.706	< 0.001*	43.28 (11.47)	57.44 (7.26)	62.92 (3.53)	41.133	< 0.001*
Social wellbeing	1.23†	2.22†	2.55†	152.827	< 0.001*	12.96 (4.87)	18.56 (5.87)	21.00 (3.20)	23.109	< 0.001*
Emotion AP	1.30†	2.13†	2.57†	157.909	< 0.001*	13.36 (4.59)	17.28 (3.47)	19.72 (0.79)	23.023	< 0.001*
Overall AP	1.42†	1.96†	2.62†	132.578	< 0.001*	69.60 (19.19)	81.11 (13.04)	90.13 (5.18)	15.816	< 0.001*
**Within control group**										
Routine Activity	1.33†	2.27†	2.40†	120.731	< 0.001*	45.96 (6.00)	55.57 (10.37)	60.04 (8.73)	20.375	< 0.001*
Social wellbeing	1.31†	1.96†	2.73†	167.376	< 0.001*	14.43 (3.81)	16.78 (4.46)	20.35 (3.11)	16.855	< 0.001*
Emotion AP	1.22†	2.38†	2.40†	151.804	< 0.001*	13.74 (3.05)	17.13 (3.71)	18.43 (2.86)	17.230	< 0.001*
Overall AP	1.47†	1.98†	2.55†	167.376	< 0.001*	74.13 (9.63)	77.81 (13.99)	85.94 (11.44)	8.621	< 0.001*

$\bar{\text{x}}$(s.d.), mean (standard deviation); χ^2^, chi-square value from Friedman’s ANOVA; AP, activity and participation.

† , Superscript denoting post-hoc comparison difference of any superscript denotes significant difference while sameness of any superscript denotes no significant difference.

## Discussion

At the conclusion of the 12-week aerobic exercise programme, our results show a significant increase in the activity and social participation level in the exercise group as compared to the non-exercisers. This shows that among PLWH diagnosed with HAND, regular physical exercise may operate as a countermeasure to cardiorespiratory-mediated activity limitation and participation restriction. Our findings are in line with those of Stessman et al. (2002), who found a separate relationship between exercise and senior men’s and women’s ease of performance on at least three out of four ADL tasks. Similarly, for both men and women, shopping convenience was associated with physical activity (Stessman et al. 2002).

People living with HIV frequently experience functional impairment, and HIV-related physical and mental disabilities have been linked to reduced exercise capacity and daily activity levels in patients (Crystal et al. 2000; Zonta et al. 2003). HIV-positive individuals may be more susceptible to poor health outcomes and cardiovascular illnesses because of a lower aerobic capacity, which may further limit physical activity (Centre for Disease Control and Prevention 2010; Johnson et al. 1990; Scott et al. 2007). In particular, work (Chernoff et al. 2010; Marquine et al. 2018) and driving (Gouse et al. 2020; Thames et al. 2011) are among the activities of daily life that are negatively correlated with neurocognitive impairment (Marquine et al. 2018; Schifitto et al. 2001).

According to Cohen, Seider and Navia (2015), HIV-related neurocognitive impairments represent a separate risk factor for accelerated ageing and frailty in people living with HIV. According to Seider et al. (2014), HIV infection is also linked to accelerated cognitive ageing, meaning that individuals in their 50s and 60s are cognitively functioning more like those in their 70s and 80s. It is plausible that the exercise group’s inability to improve their performance on routine tasks could have nothing to do with their increased heart rate or aerobic insufficiency. Though cardiorespiratory insufficiency and frailty-related changes are not uncommon findings among PLWH, especially those with HAND, we expected better cardiorespiratory fitness as measured by reduced pulse rate among the exercise group (Erlandson et al. 2018; Masters et al. 2021; Neto et al. 2016; Thames et al. 2011).

Significantly, the increases in activity and involvement did not continue for 3 months following the conclusion of the exercise, suggesting that the effects on these variables were transient. As far as we are aware, this is the first study looking at how aerobic exercise affects participation and activity levels in PLWH with HAND diagnoses. According to Sebastião et al. (2019), there is only a weak correlation between participating in exercise and instrumental daily living activities. Nevertheless, we think that the exercisers’ shown aerobic deficiency may have contributed to the brief effect of exercise that our study showed.

Remarkably, we also saw an improvement in the control group from the baseline to the follow-up, which may indicate a confounding factor that we overlooked. As the majority of study participants were receiving first-line ART, a change in medication was observed at the start of the investigation. As required by the WHO’s updated guidelines for all people, the pharmaceutical regimen was changed from Lamivudine/Tenofovir/Efavirenz combination to Lamivudine/Tenofovir/Dolutegravir combination (WHO 2018). According to reports, in non-pregnant people, dolutegravir achieves virologic control three times faster than efavirenz (Walmsley et al. 2013).

The post-randomisation exclusion may have constrained our study outcomes and undermined the purpose of randomisation. Nevertheless, the advantage of randomisation was maintained by using intention-to-treat analysis. As less than 75% of the time is regarded as unsatisfactory, our lower compliance rate represents a constraint on the study. We provided incentives and directed participants to nearby treatment facilities in an effort to improve adherence to the intervention. As their quality of life (QoL) improves, individuals with HIV are often lost to follow-up with lower treatment adherence, as other studies have shown (Dudley et al. 1995; Hessol et al. 2001). This could account for the study’s observed non-adherence rate. People with HAND have a brief increase in activity and involvement when they engage in aerobic exercise. Frequent aerobic exercise is a good way to help PLWH with HAND manage their activity and participation limitations. To determine the ideal conditions for exercise efficacy and persistence, more research is required.
